# MAP1B rescues LRRK2 mutant-mediated cytotoxicity

**DOI:** 10.1186/1756-6606-7-29

**Published:** 2014-04-22

**Authors:** Sharon L Chan, Ling-Ling Chua, Dario C Angeles, Eng-King Tan

**Affiliations:** 1 National Neuroscience Institute, SGH Campus, Singapore, Singapore; 2 Department of Neurology, Singapore General Hospital, Singapore, Singapore; 3 National Neuroscience Institute (SGH Campus) and Duke-NUS Graduate Medical School Singapore, Singhealth Research Facilities, Blk A #02-02, Singapore 169611, Singapore

**Keywords:** LRRK2, MAP1B, LC1, Phosphorylation, Apoptosis

## Abstract

Leucine-rich repeat kinase 2 (LRRK2) mutations are the most common cause of dominant and sporadic Parkinson’s disease (PD), a common neurodegenerative disorder. Yeast-two-hybrid screening using human LRRK2 kinase domain as bait identified microtubule associated protein 1B (MAP1B) as a LRRK2 interactor. The interacting domains were LRRK2 kinase and the light chain portion of MAP1B (LC1). LRRK2 + LC1 interaction resulted in LRRK2 kinase inhibition. LRRK2 mutants (R1441C, G2019S and I2020T) exhibited decreased endogenous LC1 expression and its co-expression with LC1 rescued LRRK2 mutant-mediated toxicity. This study presented the first data on the effects of LRRK2 + LC1 interaction and also suggested that LCI possibly rescued LRRK2 mutant-induced cytotoxicity by inhibiting LRRK2 kinase activity. Compounds that upregulate LC1 expression may therefore hold therapeutic potential for LRRK2-linked diseases.

## Introduction

Parkinson’s disease (PD), a neurodegenerative disorder, has been estimated to afflict six million people worldwide [1,2] and mutations in the leucine-rich repeat kinase 2 (LRRK2) gene is the most common cause of dominant and sporadic PD [3,4]. Common pathogenic LRRK2 mutations like R1441C/G, Y1699C, G2019S and I2020T reside within the Roc-COR-kinase domains and are associated with increased kinase activity, which is in turn linked to increased neurotoxicity [5,6]. Though the enzymatic functions of LRRK2 have been extensively studied, its physiological mechanism remains unknown. Hence, recent LRRK2 research focused on the identification of LRRK2 interactors/substrates as they will provide vital pathophysiologic clues.

Recently, LRRK2 was reported to phosphorylate and negatively regulate Futsch, the fly homolog of microtubule-associated protein 1B (MAP1B), at the pre-synapse [7]. The MAP1B complex comprises of the heavy chain (HC) and light chain (LC1) subunits [8]. LC1 has been reported to dimerize or oligomerize [9] and its overexpression can lead to endoplasmic reticulum stress-induced apoptosis [10].

Utilising yeast-two-hybrid screening with human LRRK2 kinase domain as bait, LC1 was identified as a LRRK2 interactor. This study presented the first data on the effects of LRRK2 and LC1 interaction and also suggested that LCI possibly rescued LRRK2 mutant-induced cytotoxicity by inhibiting LRRK2 kinase activity.

## Materials and methods

### Immunocytochemistry

SKNSH cells were fixed in 4% paraformaldehyde and probed with LRRK2 (Novus) and LC1 (Santa Cruz) primary antibodies (1:200) at 4°C overnight. Subsequently, secondary antibodies (Invitrogen), Alexa488 (LRRK2) and Alexa546 (LC1), were added to cells and incubated at room temperature for one hour. Cells were mounted with DAPI and immunofluorescence was visualised.

### Kinase assays

LRRK2 kinase assay was carried out using the ADP Hunter assay (BMG labtech, Offenburg, Germany), truncated LRRK2 protein (Invitrogen), 10 mM ATP (Sigma-Aldrich) and purified LC1-GST. Reagents were mixed and incubated at room temperature for two hours; reaction was stopped by adding sample loading dye and boiling for five minutes. Primary antibodies, phospho-threonine (Cell Signaling) and phospho-serine (Millipore) were used at the concentration 1:1000.

### Western blotting

Human LRRK2 WT, G2019S and I2020T were cloned into pEGFP-N1 vectors and LC1 was cloned into pGEX5X1 vector. R1441C plasmid was cloned by Mark Cookson [11] and obtained from Addgene (plasmid 25046). Human neuroblastoma cells (SKNSH; ATCC) was transfected using Turbofect (Thermo Scientific) and incubated for 48 hours before cell lysates were collected and resolved by SDS-PAGE. Primary antibodies, LC1 (Santa Cruz) and β-actin (Sigma-Aldrich), were used at the concentration 1:1000 and all secondary antibodies (Santa Cruz) were used at the concentration 1:2000. Resultant western blot bands were quantified using the NIH ImageJ software [12] and tabulated in a bar graph.

### Functional assays

Cell viability assays utilised Methylthiazolyldiphenyl-tetrazolium bromide (MTT, Sigma-Aldrich); MTT (0.5 mg/mL) was added to transfected cells and incubated at 37°C for three hours before resultant formazan was solubilised in DMSO and quantified. Caspase-Glo 3/7 luminescent apoptosis assay (Promega, Wisconsin, USA) was carried out according to manufacturer’s instructions. Statistical analysis was carried out using the Student’s *t*-test.

## Results

**Figure 1:**
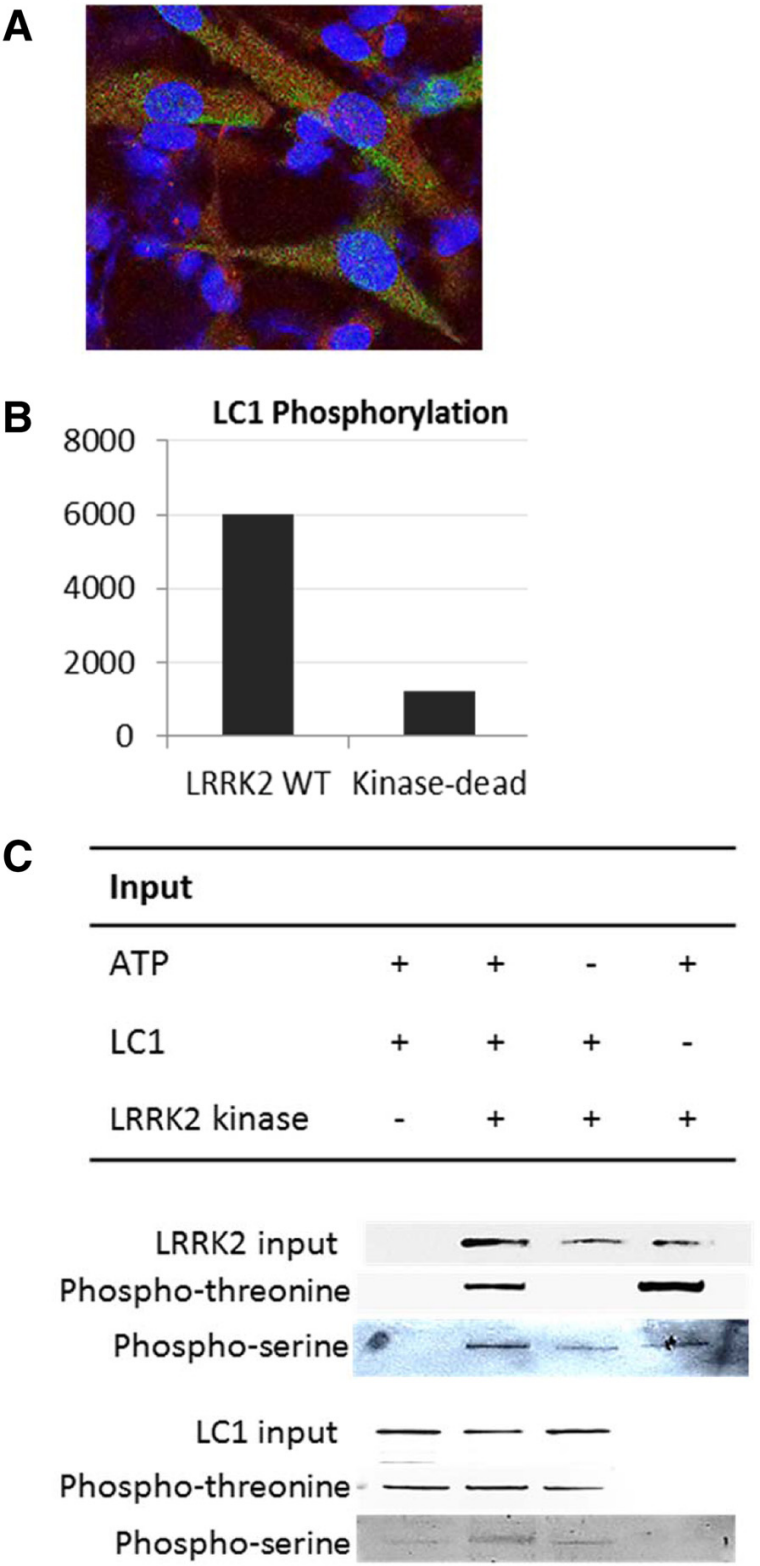
**The interaction and phosphorylation of LRRK2 and LC1. (A)** Cellular co-localisation of endogenous LRRK2 and MAP1B (LC1). SKNSH cells were labelled with LRRK2 (green) and LC1 (red) antibodies and signals were detected by immunofluorescence. Detailed figure in Additional file 1. **(B)** LRRK2 kinase assay. The LRRK2 wild-typed (WT) kinase interacted and phosphorylated the LC1 domain unlike the LRRK2 kinase-dead protein. **(C)** Western blotting of LRRK2 and LC1 proteins after a kinase assay. Both LRRK2 and LC1 proteins were probed for phospho-threonine and phospho-serine signals respectively.

Yeast-two-hybrid screening was performed as previously mentioned [13]. Briefly, a human brain cDNA library was screened using the human LRRK2 kinase domain as bait. Three out of 54 surviving clones in highly stringent growth media contained open reading frames that shared 99% homology to LC1 (accession number NM_005909; data not shown). The interaction between LRRK2 and LC1 was validated by their subcellular co-localisation in SKNSH using immunofluorescence (Figure 1A and Additional file 1: Figure S1). LRRK2 + LC1 interaction was further supported when purified LC1-GST protein successfully pulled down the LRRK2 kinase domain that was overexpressed in SKNSH (Additional file 1: Figure S2). Consequently, a LRRK2 kinase assay was carried out to reveal that LC1 was phosphorylated by LRRK2 at its serine residues but not threonine. Additionally, LRRK2 + LC1 interaction appeared to inhibit LRRK2 autophosphorylation at its threonine sites (Figures 1B & C). It should be noted that the phospho-threonine signals were much stronger than the phospho-serine signals via western blot detection.

**Figure 2:**
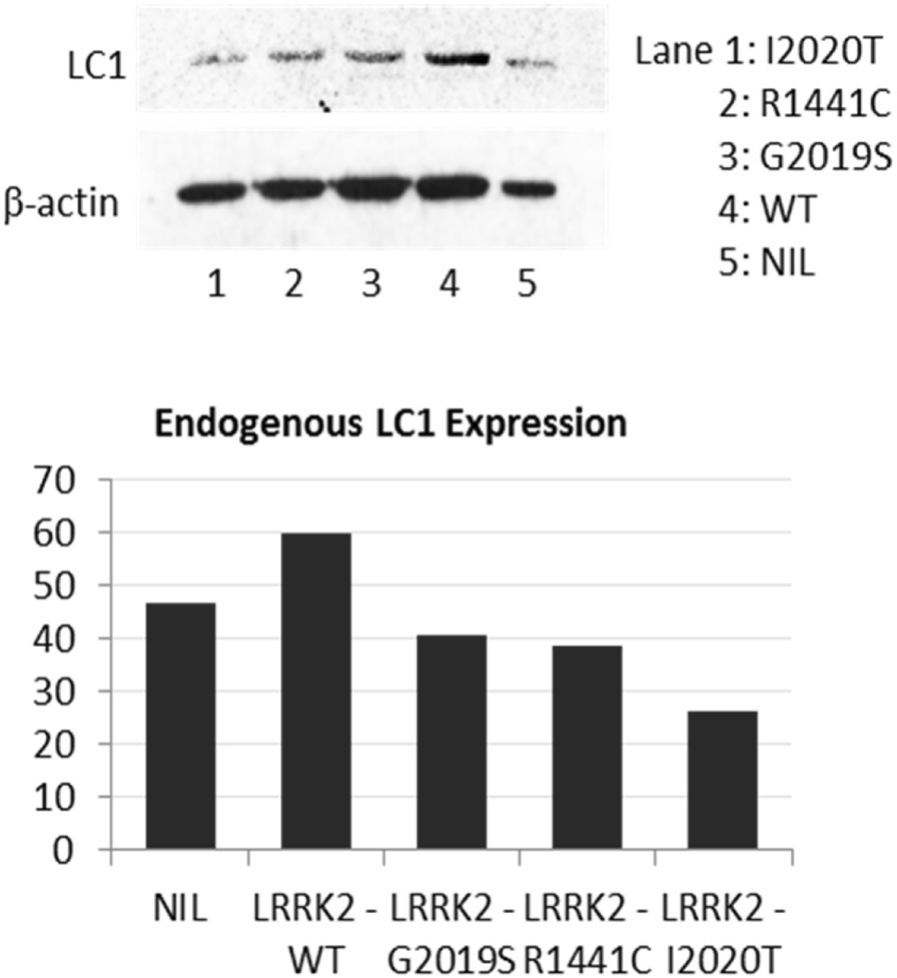
**Endogenous LC1 protein expression in SKNSH cells.** Cells were transiently expressing LRRK2-WT or one of its mutants. After 48 hours, cell lysates were analysed by western blotting and beta actin was used as a loading control. The signal intensity of the western blot bands was corrected for beta actin and quantified using the NIH ImageJ software.

To investigate the effect of LRRK2 mutations on endogenous LC1 expression, wild-typed LRRK2 (WT) or its mutants (G2019S, R1441C, I2020T) were transiently expressed in SKNSH. Cell lysates were analysed by SDS-PAGE to reveal a decrease in endogenous LC1 expression with LRRK2 mutants compared to WT (Figure 2).

**Figure 3:**
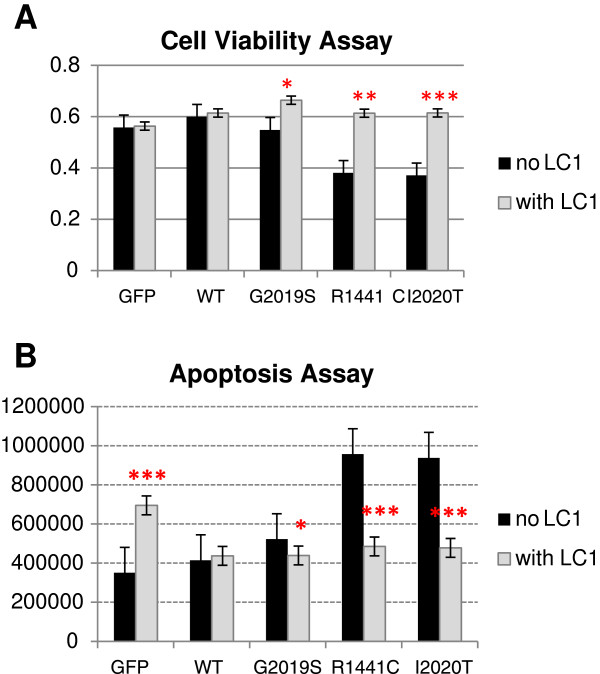
**Functional assays of LRRK2 and LC1 interaction. (A)** MTT assay of SKNSH transiently expressing LRRK2-WT or its mutants alone (black columns) or co-expression with LC1 (grey columns). Experiment was performed in triplicates and repeated three times. Data represent mean values with standard error bars. **(B)** Apoptosis assay of SKNSH transiently expressing LRRK2 or its mutants alone (black columns) or co-expression with LC1 (grey columns). Experiment was carried out with six replicates and data in the bar graph represent mean values with standard error bars. Statistical significance was determined using Student’s *t*-test and *p < 0.05, **p < 0.01 and ***p < 0.001.

As LRRK2 mutants have been shown to be more neurotoxic than WT [5], the effect of LC1 co-expression on LRRK2-mediated cytotoxicity was investigated. SKNSH transfected with LRRK2 alone or co-transfected with LRRK2 + LC1 was assayed for their cell viability using MTT. The co-expression of LRRK2 + LC1 appeared to rescue the neuronal cells from LRRK2 mutant-mediated cytotoxicity (Figure 3A). An apoptosis assay was next carried out to determine if this LC1-related rescue is linked to apoptosis. The overexpression of LC1 in control cells appeared to increase the levels of apoptosis, whereas the co-expression of LRRK2 appeared to neutralize or decrease the extent of apoptosis (Figure 3B).

## Discussion

MAP1B was identified as a LRRK2 interactor through yeast-two-hybrid screening using human LRRK2 kinase domain as bait; the interacting domains were narrowed down to the LRRK2 kinase and LC1. The MAP1B complex comprises of the HC and LC1 subunits. LC1 has microtubule stabilizing activity and the HC has been reported to act as the regulatory subunit of the MAP1B complex to control LC1 activity [9]. LC1 appeared to inhibit LRRK2 kinase activity and a decrease in endogenous LC1 expression was observed in LRRK2 mutants that exhibited increased toxicity. Subsequent functional assays showed that transient LRRK2 mutant expression caused acute toxicity through apoptosis, which could be rescued by the co-expression of LC1. LRRK2 kinase activity is central to its pathogenicity as LRRK2 kinase-dead mutants are not toxic [11]. These observations suggest that LCI is an important mediator of LRRK2 mutant-related toxicity and this is possibly linked to LRRK2 kinase inhibition.

The interaction between LRRK2 and LC1 is supported by an earlier report which showed that LRRK2 phosphorylates Futsch, a fly homolog of MAP1B [7]. LRRK2 was reported to negatively regulate the presynaptic function of Futsch in controlling microtubule dynamics [7] and this study showed that LRRK2 kinase phosphorylated LC1 at serine residues while concurrently inhibiting LRRK2 autophosphorylation at threonine residues. Similarly, as the co-expression of Futsch was able to rescue synaptic morphology defects induced by human LRRK2 overexpression [7], this report demonstrated that the co-expression of LC1 was able to neutralise or rescue the observed apoptotic effect with wild-typed LRRK2 and mutant LRRK2 respectively.

In conclusion, this study validated MAP1B as a LRRK2 interactor and specified LC1 as its interacting domain. It showed concurrent LRRK2 kinase inhibition while phosphorylating LC1 and the effect of this interaction possibly neutralised LRRK2 mutant-mediated cytotoxicity by inhibiting its kinase activity. This provided additional insight into LRRK2 mutant-mediated pathogenicity and the regulation of LRRK2+ LC1 interaction is a potential avenue for the development of LRRK2 therapeutics.

## Competing interests

The authors declare that they have no conflict of interest related to the present work.

## Authors’ contributions

SLC and ET drafted the manuscript. SLC conceived the study, carried out western blotting and functional assays; LC carried out immunofluorescence, kinase assays and cell viability assay; DCA carried out yeast-two-hybrid experiment. All authors read and approved the final manuscript.

## Supplementary Material

Additional file 1: Figure S1 Cellular co-localisation of endogenous LRRK2 and MAP1B-LC1 (LC1). Endogenous LRRK2 (green) and LC1 (red) in SKNSH were labelled with antibodies and signals were detected using immunofluorescence. Nucleus was stained with DAPI (blue) and figures were merged to observe co-localisation. **Figure S2.** LC1-GST pulldown assay. Purified LC1-GST protein and GST protein were used to pull down LRRK2 kinase myc from LRRK2 kinase myc-transfected SKNSH lysate. Purified protein was added to SKNSH lysate and incubated at 4°C for 2 hours before detecting with anti-myc antibody. Click here for file

## References

[B1] Dauer W Przedborski S Parkinson’s disease: mechanisms and models Neuron 2003 39 889 909 10.1016/S0896-6273(03)00568-3 12971891

[B2] Braak H Del Tredici K Rub U de Vos RA Jansen Steur EN Braak E Staging of brain pathology related to sporadic Parkinson’s disease Neurobiol Aging 2003 24 197 211 10.1016/S0197-4580(02)00065-9 12498954

[B3] Kumari U Tan EK LRRK2 in Parkinson’s disease: genetic and clinical studies from patients FEBS J 2009 276 6455 6463 10.1111/j.1742-4658.2009.07344.x 19804413

[B4] Wider C Dickson DW Wszolek ZK Leucine-rich repeat kinase 2 gene-associated disease: redefining genotype-phenotype correlation Neurodegener Dis 2010 7 175 179 10.1159/000289232 20197701 PMC2859237

[B5] West AB Moore DJ Choi C Andrabi SA Li X Dikeman D Biskup S Zhang Z Lim KL Dawson VL Dawson TM Parkinson’s disease-associated mutations in LRRK2 link enhanced GTP-binding and kinase activities to neuronal toxicity Hum Mol Genet 2007 16 223 232 17200152 10.1093/hmg/ddl471

[B6] Berwick DC Harvey K LRRK2 signaling pathways: the key to unlocking neurodegeneration? Trends Cell Biol 2011 21 257 265 10.1016/j.tcb.2011.01.001 21306901

[B7] Lee S Liu HP Lin WY Guo H Lu B LRRK2 kinase regulates synaptic morphology through distinct substrates at the presynaptic and postsynaptic compartments of the Drosophila neuromuscular junction J Neurosci 2010 30 16959 16969 10.1523/JNEUROSCI.1807-10.2010 21159966 PMC3045823

[B8] Hammarback JA Obar RA Hughes SM Vallee RB MAP1B is encoded as a polyprotein that is processed to form a complex N-terminal microtubule-binding domain Neuron 1991 7 129 139 10.1016/0896-6273(91)90081-A 1712602

[B9] Togel M Wiche G Propst F Novel features of the light chain of microtubule-associated protein MAP1B: microtubule stabilization, self interaction, actin filament binding, and regulation by the heavy chain J Cell Biol 1998 143 695 707 10.1083/jcb.143.3.695 9813091 PMC2148156

[B10] Wang Z Zhang Y Zhang S Guo Q Tan Y Wang X Xiong R Ding J Chen S DJ-1 can inhibit microtubule associated protein 1 B formed aggregates Mol Neurodegener 2011 6 38 10.1186/1750-1326-6-38 21645326 PMC3120713

[B11] Greggio E Jain S Kingsbury A Bandopadhyay R Lewis P Kaganovich A van der Brug MP Beilina A Blackinton J Thomas KJ Ahmad R Miller DW Kesavapany S Singleton A Lees A Harvey RJ Harvey K Cookson MR Kinase activity is required for the toxic effects of mutant LRRK2/dardarin Neurobiol Dis 2006 23 329 341 10.1016/j.nbd.2006.04.001 16750377

[B12] Schneider CA Rasband WS Eliceiri KW NIH Image to ImageJ: 25 years of image analysis Nat Methods 2012 9 671 675 10.1038/nmeth.2089 22930834 PMC5554542

[B13] Angeles DC Gan BH Onstead L Zhao Y Lim KL Dachsel J Melrose H Farrer M Wszolek ZK Dickson DW Tan EK Mutations in LRRK2 increase phosphorylation of peroxiredoxin 3 exacerbating oxidative stress-induced neuronal death Hum Mutat 2011 32 1390 1397 10.1002/humu.21582 21850687

