# Supplementary material for: MAP1B rescues LRRK2 mutant-mediated cytotoxicity
Source: Mol Brain. 2014 Apr 22;7:29. doi: 10.1186/1756-6606-7-29 (PMC4022373; doi:10.1186/1756-6606-7-29)
Supplement: Additional file 1: Figure S1 — Cellular co-localisation of endogenous LRRK2 and MAP1B-LC1 (LC1). Endogenous LRRK2 (green) and LC1 (red) in SKNSH were labelled with antibodies and signals were detected using immunofluorescence. Nucleus was stained with DAPI (blue) and figures were merged to observe co-localisation. Figure S2. LC1-GST pulldown assay. Purified LC1-GST protein and GST protein were used to pull down LRRK2 kinase myc from LRRK2 kinase myc-transfected SKNSH lysate. Purified protein was added to SKNSH lysate and incubated at 4°C for 2 hours before detecting with anti-myc antibody. [file 1756-6606-7-29-S1.docx]

**Additional file 1**


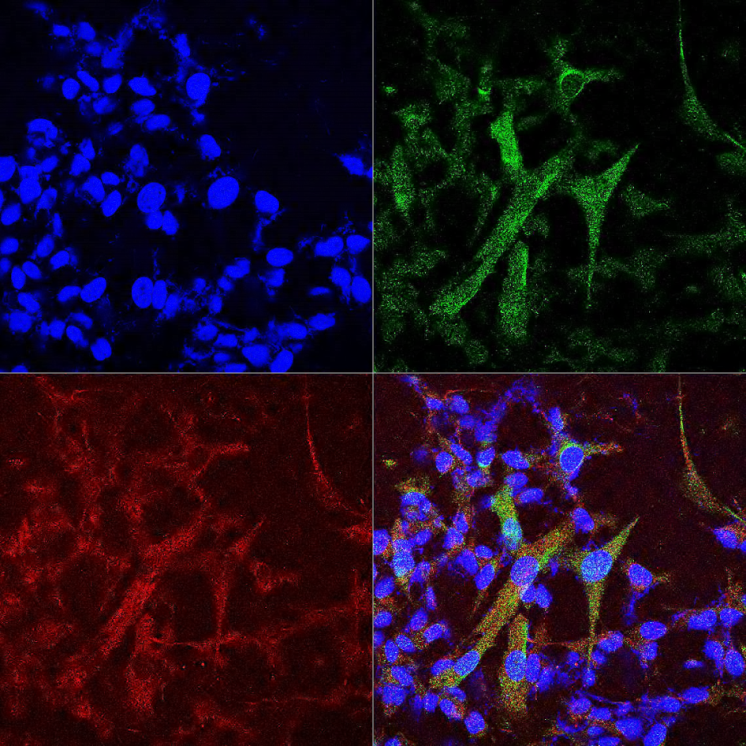


**Figure S1. Cellular co-localisation of endogenous LRRK2 and MAP1B-LC1 (LC1).** Endogenous LRRK2 (green) and LC1 (red) in SKNSH were labelled with antibodies and signals were detected using immunofluorescence. Nucleus was stained with DAPI (blue) and figures were merged to observe co-localisation.


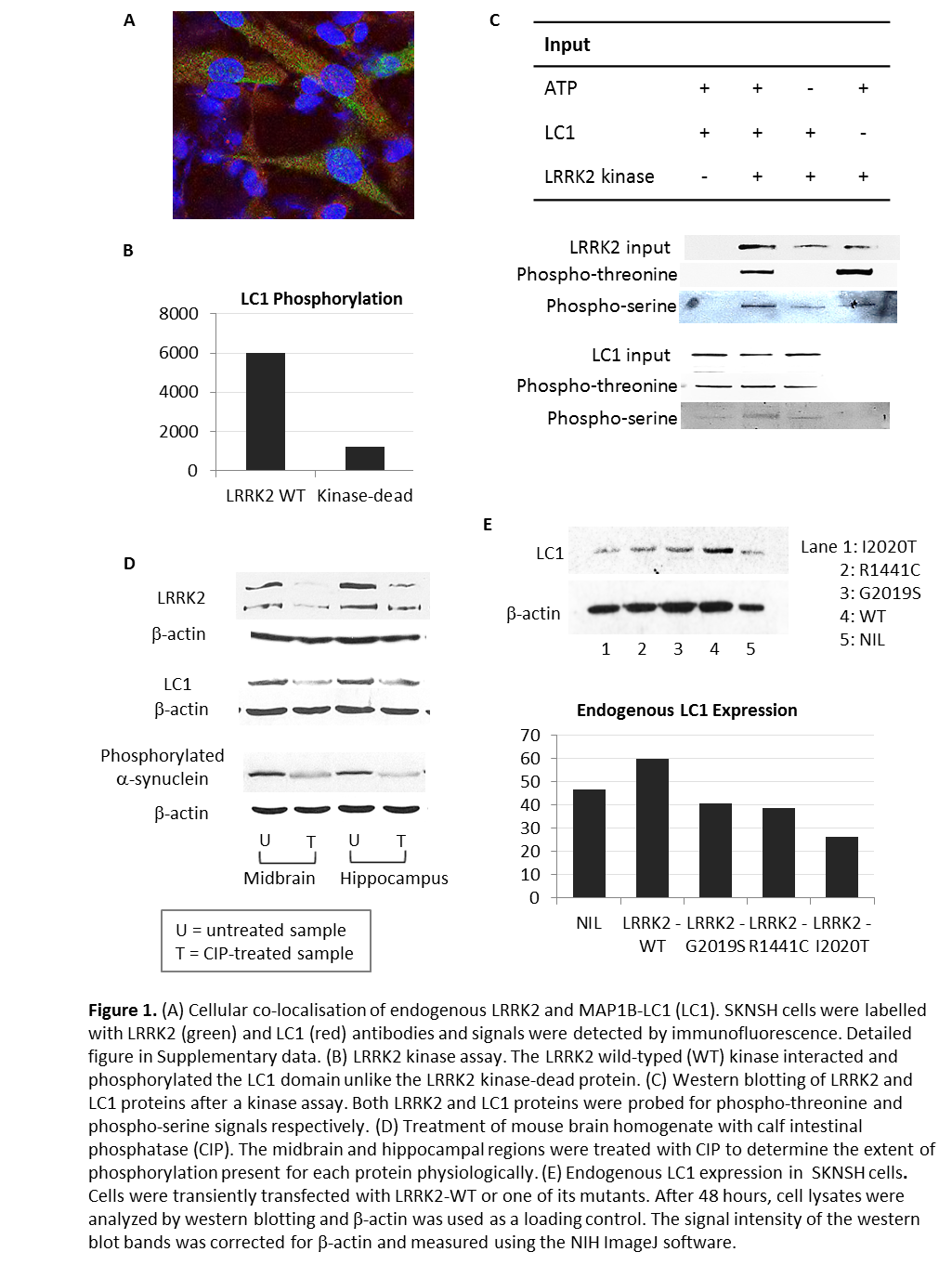

**Figure S2. LC1-GST pulldown assay.** Purified LC1-GST protein and GST protein were used to pull down LRRK2 kinase myc from LRRK2 kinase myc-transfected SKNSH lysate. Purified protein was added to SKNSH lysate and incubated at 4^o^C for 2 hours before detecting with anti-myc antibody.
